# Diagnostic significance of reassessment of prostate biopsy specimens by experienced urological pathologists at a high-volume institution

**DOI:** 10.1007/s00428-022-03272-0

**Published:** 2022-01-11

**Authors:** Yoichiro Okubo, Yayoi Yamamoto, Shinya Sato, Emi Yoshioka, Masaki Suzuki, Kota Washimi, Kimito Osaka, Takahisa Suzuki, Tomoyuki Yokose, Takeshi Kishida, Yohei Miyagi

**Affiliations:** 1 Department of Pathology, Kanagawa Cancer Center, 2-3-2, Nakao, Asahi-Ku, Yokohama, Kanagawa 241-8515 Japan; 2 Department of Radiology, Kanagawa Cancer Center, 2-3-2, Nakao, Asahi-Ku, Yokohama, Kanagawa 241-8515 Japan; 3 Molecular Pathology and Genetics Division, Kanagawa Cancer Center Research Institute, 2-3-2, Nakao, Asahi-Ku, Yokohama, Kanagawa 241-8515 Japan; 4 Department of Pathology, University of Tokyo Institute, 7-3-1 Hongo, Bunkyo-Ku, Tokyo, 113-8655 Japan; 5 Department of Urology, Kanagawa Cancer Center, 2-3-2, Nakao, Asahi-Ku, Yokohama, Kanagawa 241-8515 Japan

**Keywords:** Prostate, Adenocarcinoma, Gleason score, Grade group, Biopsy

## Abstract

In prostate cancer, accurate diagnosis and grade group (GG) decision based on biopsy findings are essential for determining treatment strategies. Diagnosis by experienced urological pathologists is recommended; however, their contribution to patient benefits remains unknown. Therefore, we analyzed clinicopathological information to determine the significance of reassessment by experienced urological pathologists at a high-volume institution to identify factors involved in the agreement or disagreement of biopsy and surgical GGs. In total, 1325 prostate adenocarcinomas were analyzed, and the GG was changed in 452/1325 (34.1%) cases (359 cases were upgraded, and 93 cases were downgraded). We compared the highest GG based on biopsy specimens, with the final GG based on surgical specimens of 210 cases. The agreement rate between the surgical GG performed and assessed in our institute and the highest biopsy GG assessed by an outside pathologist was 34.8% (73/210); the agreement rate increased significantly to 50% (105/210) when biopsy specimens were reevaluated in our institute (chi-square test, *P* < 0.01). Multivariate logistic regression analysis showed that only the length of the lesion in the positive core with the highest GG in the biopsy was a significant factor for determining the agreement between biopsy GG and surgical GG, with an odds ratio of 1.136 (95% confidence interval: 1.057–1.221; *P* < 0.01). Thus, reassessment by experienced urological pathologists at high-volume institutions improved the agreement rate. However, it should be noted there is a high probability of discordance between a small number of lesions or short lesions and surgical GG.

## Introduction

The incidence of prostate cancer is on the rise worldwide [1, 2]. There are various treatment options available, including hormonal therapy, radiation, carbon-ion radiotherapy, and radical prostatectomy [3–5]. Along with clinical information, including serum prostate-specific antigen (PSA) levels and imaging findings, histopathological diagnosis based on prostate needle core biopsy results is important to provide the most appropriate treatment [6–8]. A biopsy requires the determination of Gleason score (GS) or grade group (GG), presence/absence of cancer, and histological type. Particularly, the highest GG in a positive core has a significant impact on treatment strategy [9–11]. For example, if the serum PSA level is low and the patient has GG1, active surveillance is recommended [12]. Patients with GG4 or higher are classified as high risk for D'Amico classification based on histology alone, and extended pelvic lymph node dissection is considered [13]. However, prostate needle biopsies are not always performed at the treating institution and are sometimes performed at the referring institute. At smaller facilities, the diagnosis is not always made by experienced pathologists. Although diagnosis by experienced urological pathologists provides a more accurate GG assessment than inexperienced pathologists [14–16], only a few cases are available to verify whether reassessments are truly beneficial for the patient [17]. As our institution is a high-volume institution with experienced urological pathologists, we often reassess specimens from other institutes. In this study, we sought to determine the significance of reassessment at a high-volume institution and the factors involved in the agreement or disagreement between the GG based on the prostate needle core biopsy results and that based on surgical specimens.

## Materials and methods

### Study design and population

Patients who were referred to our institute between November 2018 and September 2021, and who underwent reassessment of prostate needle core biopsy results taken at another institute, were included in this retrospective study. All biopsy specimens were reassessed by our pathologists.

### Data collection

In addition to reassessment, the following parameters were collected by confirming the pathology request form and electronic medical records and contacting the referring institute: age, serum PSA level, histological diagnosis, GG determined by outside pathologists, reassessed highest GG at our institute, number of biopsies obtained, number of positive cores, lesion length of the highest GG, and history of hormone therapy. Global scoring should be adopted if it could be strictly determined that the biopsy was from the same area, but in this study, biopsies were obtained at outside institutes, and specimen preparation methods vary from institution to institution. Although most sampling areas could be confirmed from reports provided by outside institutions, this was not always the case. Further, few institutes conducted multiple biopsies from the same lesion, so we decided to adopt the highest GG among the individual positive cores. For tertiary patterns, we followed the guidance of the 2019 International Society of Urological Pathology Consensus Conference [18] to include tertiary high grade patterns, regardless of percentage, in GG (e.g., a needle biopsy with 70% Gleason pattern 4, 27% pattern 3, and 3% pattern 5 would be reported as GS 4 + 5 = 9; GG5). Unfortunately, many pathologists who do not specialize in urology are unfamiliar with evaluations of prostate intraductal carcinoma (IDC-P), and therefore, most reports from outside institutes contained no IDC-P results. We referred to previous reviews [19] and excluded IDC-P lesion from the GG assessment in this study. We also confirmed whether the reassessed patients had subsequently undergone radical prostatectomy at our institute. In the case of multiple lesions in a surgical specimen, the lesion with the highest GG was included in the analyses. We also collected data on changes in lymph node dissection criteria with changes in GG.

### Assessment of the biopsy and surgical specimens

Biopsy GG decisions were made independently by the pathologists (YO, EY, MS, and KW); in case of uncertainty, two or more pathologists discussed their opinions. The GG was assigned precisely according to the World Health Organization Classification of Tumours of the Urinary System and Male Genital Organs [20], which perfectly reflects the latest consensus of a prostate cancer grading conference held in 2014 in Chicago by The International Society of Urological Pathology [21]. If there was still disagreement, the decision of YO, who had diagnosed more prostate biopsies, was given priority (15 years of experience). For surgical specimens, the first pathologist (YO or SS) described the primary pathology findings, and the specimens were reviewed by a second pathologist (YM) using a multi-viewing biological microscope. In case of disagreement, the three pathologists discussed the various diagnostic findings; however, when consensus was not reached, priority was given to the expert opinion of YM who had the longest history of prostate cancer diagnoses (over 30 years of experience).

### Statistical analyses

The chi-square test was used to compare the agreement between the highest GG based on the preoperative biopsy finding (the original highest GG by the outside pathologist and the reassessed highest GG at our institute) and the final GG based on the surgical specimen finding. Furthermore, the statistical relationship between the reassessed highest GG at our institute and the final GG based on the surgical specimen was determined using the adjusted residuals. We considered the adjusted residuals to be significantly different at ± 1.96; we interpreted them as tending towards higher agreement at ≥  + 1.96 and lower agreement at ≤  − 1.96. To statistically evaluate the differences between our and outside diagnoses, we also used Cohen’s weighted kappa coefficients with quadratic weights to analyze the agreement rate of GG between biopsy and surgery. Scores nearer to 1 were considered to have a higher statistical agreement. Further, multivariate logistic regression analysis was performed to extract the factors related to the agreement between the highest GG based on preoperative biopsy findings and the GG based on surgical specimen findings. The dependent variable was the agreement or disagreement between the highest GG based on preoperative biopsy findings and surgical specimen findings. Explanatory variables included age, serum PSA level, biopsy GG, number of biopsies obtained, number of positive cores, number of positive cores that had the highest GG, and length of the positive core that was the highest GG (or longest lesion if there was more than one). Statistical significance was set at *P* < 0.05. Statistical analyses were performed using IBM SPSS Statistics (IBM Corp., Armonk, NY, USA). Hormone-treated cases and cases other than adenocarcinoma were excluded as missing values.

## Results

### Overall findings

**Fig. 1:**
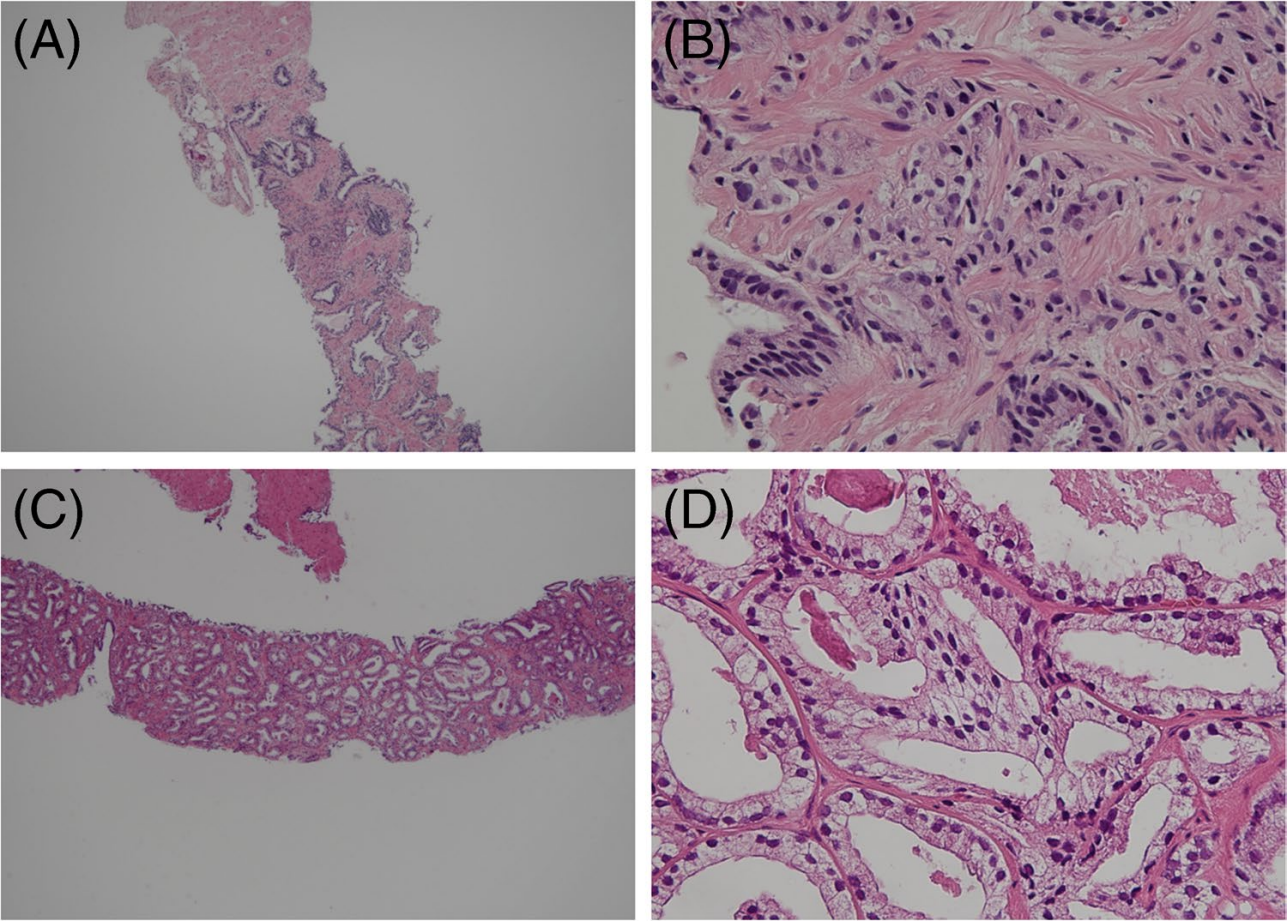
Representative cases of missed lesions and grade group changes. **A** Low-power field view of a case with a missed lesion. A lesion of only 0.7 mm is identified, which at first sight seemed to be an inflammatory cell infiltration (hematoxylin and eosin (HE) staining, × 40). **B** High-power field view shows fused glands with irregular nuclei and clear cytoplasm (HE staining, × 400). **C** Low-power field view of cases upgraded from grade group (GG)1 to GG2; most tumor areas correspond to Gleason pattern 3 (HE staining, × 40). **D** Low-power field view shows a few fused glands. In the case of needle core biopsy, even if the high grade is < 5%, it will be adopted as a secondary score. However, there are a certain number of diagnoses that were presumed to be unaware of this fact (HE staining, × 400)

We reassessed 1334 cases of prostate needle core biopsy obtained from outside institutes between January 2018 and September 2021. In four cases, the diagnosis was changed to an atypical gland because it was difficult to identify adenocarcinoma; two cases were changed to small cell carcinoma, two to sarcoma, and one to prostatic invasion of urothelial carcinoma. Of the remaining 1325 cases, 248 (18.7%) received radical prostatectomy at our institution, 36 received preoperative hormone therapy after biopsy, and 2 had prostate sarcoma (Table 1). For the remaining 210 cases, we compared the highest GG based on preoperative biopsy findings (the original highest GG by outside pathologists and the reassessed highest GG at our institute) with the final GG based on surgical specimen findings. The agreement rate between the original highest GG by outside pathologists and the surgical GG was 34.8% (73/210), whereas that between reassessed highest GG at our institute and the surgical GG was 50% (105/210); there was a significant increase in the agreement rate (chi-square test, *P* < 0.01). In 79/1325 (6%) cases, a carcinoma lesion was missed. The average length of the missed lesions was 0.71 mm (Table 1, Fig. 1A, B); overall, the average lesion length in 63/79 (79.7%) cases was < 1 mm and < 2 mm in 74/79 (93.7%) cases. In addition, missed positive core resulted in a change of the highest GG in only one case.

**Table 1:** Detailed information for the 1325 cases of prostate biopsies obtained at other institutions

Age (years, mean ± SD)	70.4 ± 7.3
Serum PSA value (ng/mL, mean ± SD)	24.5 ± 114.2
Highest GG by outside pathologists (cases and percentage)	GG1 (188, 14.2%), GG2 (339, 25.6%), GG3 (234, 17.7%), GG4 (378, 28.5%), and GG5 (186, 14.0%), respectively
Highest GG reassessed at our institute (cases and percentage)	GG1 (110, 8.3%), GG2 (289, 21.8%), GG3 (262, 19.8%), GG4 (463, 34.9%), and GG5 (201, 15.2%), respectively
Number of biopsies obtained (mean ± SD)	13.3 ± 3.5
Number of positive cores (mean ± SD)	4.6 ± 3.3
Number of highest GG cores (mean ± SD)	2.4 ± 2.2
Lesion length of the highest GG (mm, mean ± SD)	6.3 ± 4.5
Number of missed cases	79 (6%, 79/1325)
Lesion length of the missed cases (mm, mean ± SD)	0.68 ± 0.71

Abbreviations: SD, standard deviation; PSA, prostate-specific antigen; GG, grade group

Patient background and detailed biopsy information is included for 1325 cases, excluding nine cases other than adenocarcinoma from the total 1334 cases

### Reassessment results

In 873/1325 (65.9%) cases, the original highest GG and the reassessed highest GG at our institute were in agreement. Among the 873 patients, 159 received radical prostatectomy at our institute, whereas 29 received hormone therapy after biopsy, which precluded a comparison of the GGs. In the remaining 130 cases, the agreement between the highest biopsy GG and the surgical GG was 49.2% (64/130).

In 452/1325 (34.1%) cases, the original highest GG by outside pathologists and the reassessed highest GG at our institute were not in agreement. Of these cases, 359 (79.4%) had an upgraded GG upon reassessment at our institute comprising 79 (22.0%), 109 (30.4%), 89 (24.8%), 31 (8.6%), and 51 (14.2%) GG1 to GG2, GG2 to GG3, GG3 to GG4, GG4 to GG5, and others (two or more upgrades), respectively. Among these, 70 patients received radical prostatectomy at our institute, and 5 received hormone therapy after biopsy. For the remaining 65 cases, the agreement between the highest GG at our institute and the surgical GG was significantly higher: 8/65 (12.3%) for the original highest GG by outside pathologists compared with 32/65 (49.2%) for the reassessed highest GG at our institute (chi-square test, *P* = 0.003). A representative case is shown in Fig. 1C, D.

In contrast, 93 (20.6%) patients had a downgraded GG on reassessment at our institute comprising 27 (29.0%), 38 (40.9%), 13 (14%), 3 (3.2%), and 12 (12.9%) GG5 to GG4, GG4 to GG3, GG3 to GG2, GG2 to GG1, and others (two or more downgrades), respectively. Among these, 17 cases received radical prostatectomy at our institute, and 2 received hormone therapy after biopsy. For the remaining 15 cases, the agreement between the highest GG at our institute and the surgical GG was higher: 1/15 (6.7%) for the original highest GG by outside pathologists compared with 8/15 (60%) for the reassessed highest GG at our institute. Because of the small number of cases, no statistically significant difference was found (chi-square test, *P* = 0.205).

### Relationship between the highest preoperative biopsy grade group and surgical grade group

In 105 of 210 cases, preoperative biopsies and surgical GGs were in agreement; in 52 cases, the surgical GG was higher than the preoperative biopsy GG; and in 53 cases, the surgical GG was lower than the preoperative biopsy GG. Regarding the 105 cases where surgical and preoperative biopsy GGs were in agreement, GG1, GG2, GG3, GG4, and GG5 accounted for 2 (1.9%), 41 (39%), 27 (25.7%), 24 (22.9%), and 11 cases (10.5%), respectively. Upgraded surgical GG cases comprised 10 (19.2%), 14 (26.9%), 6 (11.5%), 13 (25%), and 9 (17.3%) GG1 to GG2, GG2 to GG3, GG3 to GG4, GG4 to GG5, and others (two or more upgrades), respectively. Meanwhile, downgraded surgical GG cases included 1 (1.9%), 30 (56.6%), 12 (22.6%), 0 (0%), and 10 (18.9%) cases of GG5 to GG4, GG4 to GG3, GG3 to GG2, GG2 to GG1, and others (two or more downgrades), respectively. The relationship between the highest GG based on preoperative biopsy findings and the GG based on surgical specimen findings was confirmed using adjusted residual chi-square values, with − 2.9 for GG1, 3.7 for GG2, 1 for GG3, − 3.9 for GG4, and 2.2 for GG5 (Table 2).

**Table 2:** Relationship between the highest GG based on the prostate needle core biopsy finding and the final GG based on the surgical specimen finding

	Cases with GG1 based on the surgical specimen finding	Cases with GG2 based on the surgical specimen finding	Cases with GG3 based on the surgical specimen finding	Cases with GG4 based on the surgical specimen finding	Cases with GG5 based on the surgical specimen finding
Highest GG1 based on the prostate needle core biopsy finding (*n* = 15)	2	10	3	0	0
Highest GG2 based on the prostate needle core biopsy finding (*n* = 58)	0	41	14	3	0
Highest GG3 based on the prostate needle core biopsy finding (*n* = 48)	0	12	27	6	3
Highest GG4 based on the prostate needle core biopsy finding (*n* = 75)	0	8	30	24	13
Highest GG5 based on the prostate needle core biopsy finding (*n* = 14)	0	1	1	1	11
Adjusted residual (chi-square test)	-2.9	3.7	1	-3.9	2.2

Abbreviations: SD, standard deviation; PSA, prostate-specific antigen; GG, grade group

Patient background and detailed biopsy information is included for 1325 cases, excluding nine cases other than adenocarcinoma from the total 1334 cases

Comparison in Cohen’s weighed kappa coefficient with quadratic weights.

The kappa score between the original highest GG by outside pathologists and the surgical GG was 0.507 (95% confidence interval: 0.411–0.602; *P* < 0.01), whereas that between the reassessed highest GG at our institute and the surgical GG was 0.644 (95% confidence interval: 0.562–0.727; *P* < 0.01).

### Multivariate logistic regression analysis results

Only the length of the lesion in the positive core with the highest GG based on preoperative biopsy findings was a significant factor in agreement between the GG based on preoperative biopsy findings and the GG based on surgical specimen findings. The odds ratio was 1.136 (95% confidence interval: 1.057–1.221; *P* < 0.01).

### Impact of highest group grade core numbers and lesion length in preoperative biopsies

**Fig. 2:**
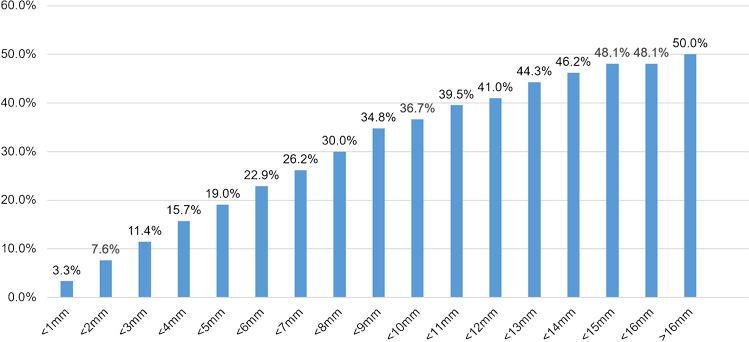
Agreement rate between the grade groups (GGs) based on the prostate needle core biopsy and surgical specimen findings for each length of the highest biopsy GG. The agreement rate increases as the lesion length in the core with the highest GG becomes longer and does not reach a plateau. It is noteworthy that the agreement rate is only 3.3% when the size is < 1 mm

The agreement rate between the lesion length of the highest GG core based on preoperative biopsies and surgical specimen findings ranged between 3.3% at < 1 mm and 36.7% at < 10 mm. The agreement increased with increasing length and tended not to reach a plateau (Fig. 2).

**Fig. 3:**
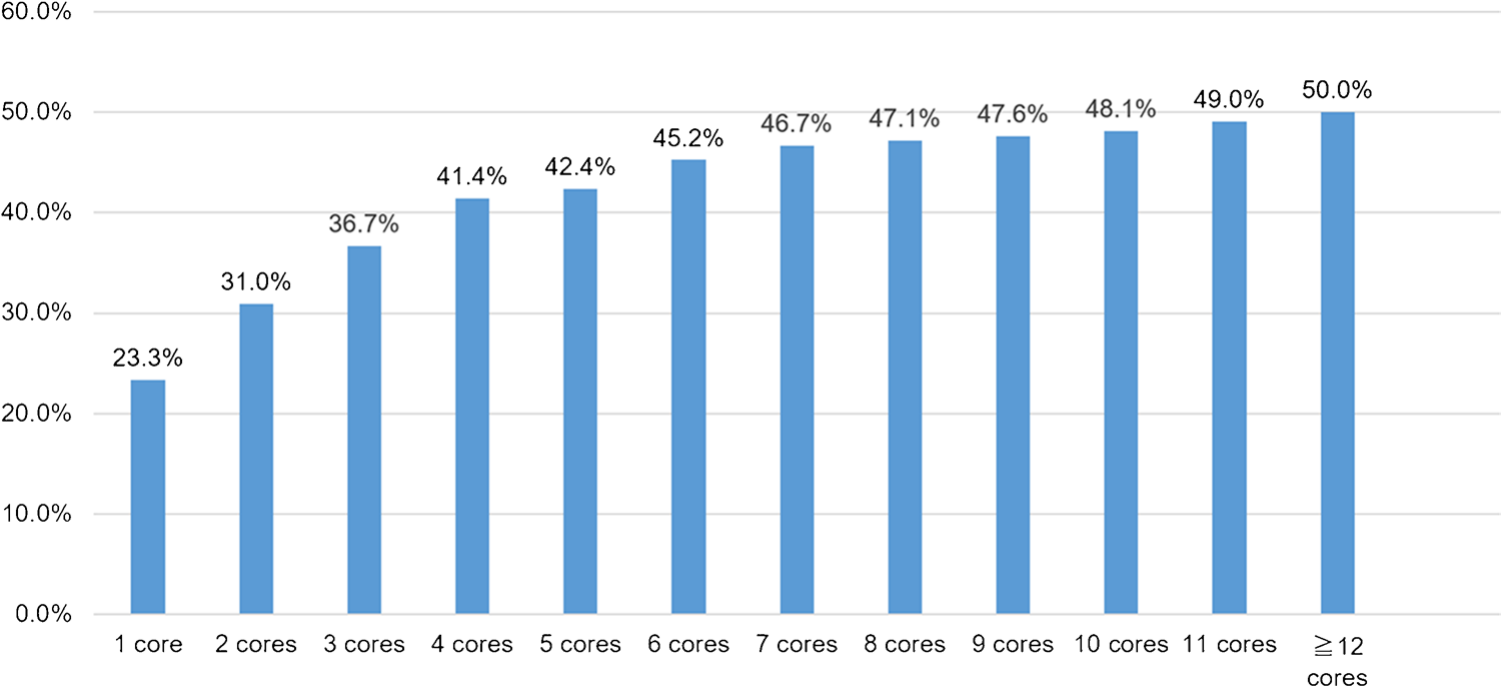
Agreement rate between grade groups (GGs) based on the prostate needle core biopsy and surgical specimen findings for each number of highest GG cores. A single positive core of the highest GG based on the preoperative biopsy finding has an agreement rate of 23.3% with the GG based on the surgical specimen finding, whereas six cores have an agreement rate of 45.2%. The higher the number of cores, the higher the agreement, but after six positive cores, the agreement increases slowly

A single positive core of the highest GG based on preoperative biopsy findings had an agreement rate of 23.3% with the GG based on surgical specimen findings, whereas six cores had an agreement rate of 45.2%. The higher the number of cores, the higher the agreement; however, after six positive cores, the agreement increased slowly (Fig. 3).

### Impact of grade group change on lymph node dissection

At our institution, lymph node dissection is conducted for patients with high risk according to the D'Amico classification or those with a predicted lymph node metastasis rate of ≥ 7% on the Briganti 2012 nomogram [22]. Following reassessment, the above criteria were met in 54 of 210 (25.7%) patients who underwent radical prostatectomy, and lymph node metastasis was confirmed in five of these patients (5/54; 9.3%). Overall, 117 of 210 patients (55.7%) underwent lymph node dissection. Of these, lymph node metastasis was found in 13.7% (16/117). If reassessment had not been performed, 63 patients would have undergone lymph node dissection, and 11 would have been diagnosed with lymph node metastasis (17.5%, 11/63). There were three cases in which the above criteria were not met by reassessment and no lymph node dissection was done.

## Discussion

Pre-treatment biopsy assessment is essential for determining appropriate treatment strategies for prostate cancer [23, 24]. Although the GG is an excellent scoring system, it is also a subjective assessment by pathologists [25, 26]; therefore, it is better for assessments to be performed by experienced urological pathologists for a more accurate diagnosis [15, 27–29]. However, it is unclear whether reassessment with experienced urological pathologists would be beneficial for patients. This study examined the significance of this approach. Reassessment of pre-treatment biopsies showed that in approximately one-third of the cases, the original highest GG by an outside pathologist did not agree with the highest GG reassessed in our institute. Approximately 80% of the disagreements were upgraded, with GG2 to GG3 as the most common upgrade, followed by GG3 to GG4 and GG1 to GG2. Overall, the assessments of other institutes tended to overestimate atypical gland ducts corresponding to GS3 and underestimate lesions corresponding to GS4, which may have led to the disagreements noted in this study. In contrast, approximately 40% of downgraded cases were from GG4 to GG3, and 30% were from GG5 to GG4. Because downgraded cases only accounted for 20% of all cases with changed GG, the limited number of cases should be considered; however, we also found that some institutes had a tendency of diagnosing GG5 and GG4.

We compared the highest GG based on preoperative biopsy findings with the GG based on surgical specimen findings; overall, we found that the agreement rate for the GG evaluated by outside pathologists was only approximately one-third, whereas that by experienced urological pathologists at our institute was approximately one-half. In particular, our reassessment improved the agreement rate by a factor of approximately 1.5. Noteworthily, in cases where the reassessed GG was upgraded or downgraded, the agreement rate between our reassessed GG and the surgical GG was approximately 50%, whereas that between outside assessed GG and the surgical GG was only approximately 10%. Although most patients had an upgraded GG following reassessment, the agreement rate of the GG based on surgical specimen findings was almost the same regardless of an upgrade or a downgrade (approximately 50%), demonstrating that an appropriate assessment was performed. The possibility of bias should be considered because assessments were performed within the same institution. However, pathologists who assessed the biopsies and surgical specimens were not always the same; therefore, a certain level of objectivity can be expected. Furthermore, statistical analysis using Cohen’s weighted kappa coefficients showed that the agreement rate statistically increased with our reassessment. Nevertheless, it should be noted that the agreement rate between the highest GG based on biopsy specimen findings and the GG based on surgical specimen findings was only approximately half even at our high-volume institution. All relevant staff (urologists, radiologists, and pathologists) should be aware of this rate. Missing cases were found in approximately 1/20 cases; most of these were lesions < 2 mm, and the highest GG was rarely changed. Although it depends on the burden of the pathologist, focusing on the GG assessment of positive cores rather than on negative cores during reassessment would be more beneficial. However, this result implies that an experienced urological pathologist may detect minimal lesions, indicating diagnosis by a urological pathologist might better prevent small lesions from being missed. Additionally, considering the burden on the pathologist performing the reassessment, it would be desirable to establish a system where not only the request form but also the report by outside pathologists is included, so the pathologist can determine the core containing the number of lesions.

In cases with upgraded GGs based on surgical specimen findings, preoperative biopsy GG3 became GG4 in approximately 10% of cases, which was slightly lower but not overly different than that of the other categories, and was presumably a reflection of the heterogeneity of prostate cancer. Contrastingly, in cases with a downgraded GG, preoperative biopsy GG4 became surgical GG3 in approximately 60% of cases, which is a large number. Because this study was based on the highest GG according to biopsy findings, we assumed that the positive core was influenced by the fact that only the sections corresponding to Gleason pattern 4 were obtained.

In terms of reassessment benefits, if lymph node dissection is applied following only original GGs from outside pathologists, 63 out of 210 cases were subjected, and lymph node metastasis was confirmed in 11/63 (17.5%). However, after our reassessment, 54 cases were subjected to additional lymph node dissection, and 5/54 (9.3%) were found to have lymph node metastasis. Namely, lymph node metastasis was detected in approximately 10% of cases of additional lymph node dissection by our reassessment. Further follow-up is required, but we believe that a lymph node metastasis rate of 10% should not be ignored, as this may speak to the reasonableness of our reassessment. Contrarily, there were only three cases that no longer met the criteria for lymph node dissection even though the biopsy was reassessed for downgrade. As serum PSA levels and imaging findings are considered in the risk classification, the impact of downgrading was small in lymph node dissections. However, operations for many cases were not performed at our institute; hence, it is necessary to carefully follow up on the clinical significance of downgraded cases.

We conducted a statistical analysis to identify predictive factors for GG in surgical specimens based on preoperative biopsies. The results of the chi-square test and adjusted residuals showed that the highest GG of GG1 based on preoperative biopsies was significantly upgraded according to surgical specimen findings, and the highest GG of GG4 was significantly downgraded. It should be recognized that if the highest GG in the preoperative biopsy is GG1 or GG4, then the GG in the surgical specimen is likely to vary.

We conducted a multivariate analysis to determine which information obtained from preoperative biopsy findings contributed to the GG based on surgical specimen findings and found that only lesion length in the positive core with the highest GG is an independent significant factor. However, whether lesion length is the only truly significant factor requires further investigation with a larger number of cases. We also investigated the relationship among the number of positive cores, lesion length, and agreement rate. Interestingly, the number of cores reached a plateau after 5–6 cores, whereas the lesion length did not reach a distinct plateau. One study suggested performing a prostate biopsy to obtain as many cores as possible [30]; however, considering our results, excessive biopsy may be unnecessary if the number of positive cores can be efficiently obtained. Notably, the agreement rate for a single highest GG is generally only one-fourth, and only 3.3% when the lesion length of the highest GG is < 1 mm. All relevant staff should be aware that GG discrepancies are very high when the number of lesions is small or the lesion length is short, and they are required to determine a treatment strategy based on this assumption.

## Limitations

This study had some limitations. Firstly, although we reassessed specimens previously assessed by outside pathologists, we did not collect information on whether these outside institutes were high-volume institutions and whether the outside pathologists were experienced in urology. Secondly, we could not confirm the biopsy methods, targeting, and preoperative image processing used at outside institutes. Nevertheless, as most outside institutes have fewer cases of prostate cancer than our institute, this study verified the significance of diagnosis by experienced urological pathologists at a high-volume institution on patient benefit.

## Conclusions

Since reassessment by experienced urological pathologists at a high-volume institution increases the agreement between the highest GG based on the preoperative biopsy finding and the final GG based on the surgical specimen finding by a factor of approximately 1.5, it is desirable to reassess actual specimens unless it is excessively burdensome for pathologists. Moreover, it should be noted that there is a high probability of discordance between a small number of short lesions and the surgical GG.

## Data Availability

The datasets used and/or analyzed during the current study are available from the corresponding author upon reasonable request.

## References

[1] Rawla P (2019). Epidemiology of prostate cancer. World J Oncol.

[2] Sung H, Ferlay J, Siegel RL, Laversanne M, Soerjomataram I, Jemal A, Bray F (2021). Global Cancer Statistics 2020: GLOBOCAN estimates of incidence and mortality worldwide for 36 cancers in 185 countries. CA Cancer J Clin.

[3] McKay RR, Feng FY, Wang AY, Wallis CJD, Moses KA (2020). Recent advances in the management of high-risk localized prostate cancer: local therapy, systemic therapy, and biomarkers to guide treatment decisions. Am Soc Clin Oncol Educ Book.

[4] Marvaso G, Corrao G, Zaffaroni M, Pepa M, Augugliaro M, Volpe S, Musi G, Luzzago S, Mistretta FA, Verri E, Cossu Rocca M, Ferro M, Petralia G, Nole F, De Cobelli O, Orecchia R, Jereczek-Fossa BA (2021). Therapeutic sequences in the treatment of high-risk prostate cancer: paving the Way towards multimodal tailored approaches. Front Oncol.

[5] Vanneste BG, Van Limbergen EJ, van Lin EN, van Roermund JG, Lambin P (2016). Prostate cancer radiation therapy: what Do clinicians have to know?. Biomed Res Int.

[6] Counago F, Sancho G, Catala V, Hernandez D, Recio M, Montemuino S, Hernandez JA, Maldonado A, Del Cerro E (2017). Magnetic resonance imaging for prostate cancer before radical and salvage radiotherapy: what radiation oncologists need to know World. J Clin Oncol.

[7] Dutta SW, Alonso CE, Libby B, Showalter TN (2018). Prostate cancer high dose-rate brachytherapy: review of evidence and current perspectives. Expert Rev Med Devices.

[8] Mano R, Eastham J, Yossepowitch O (2016). The very-high-risk prostate cancer: a contemporary update. Prostate Cancer Prostatic Dis.

[9] Srigley JR, Delahunt B, Samaratunga H, Billis A, Cheng L, Clouston D, Evans A, Furusato B, Kench J, Leite K, MacLennan G, Moch H, Pan CC, Rioux-Leclercq N, Ro J, Shanks J, Shen S, Tsuzuki T, Varma M, Wheeler T, Yaxley J, Egevad L (2019). Controversial issues in Gleason and International Society of Urological Pathology (ISUP) prostate cancer grading: proposed recommendations for international implementation. Pathology.

[10] Yoo S, Suh J, Park J, Cho MC, Son H, Jeong H (2019). Proportion of cores with the highest Gleason grade group among positive cores on prostate biopsy: does this affect the probability of upgrading or downgrading?. Scand J Urol.

[11] Fine SW, Meisels DL, Vickers AJ, Al-Ahmadie H, Chen YB, Gopalan A, Sirintrapun SJ, Tickoo SK, Reuter VE (2020). Practice patterns in reporting tertiary grades at radical prostatectomy: survey of a large group of experienced urologic pathologists. Arch Pathol Lab Med.

[12] Lonergan PE, Jeong CW, Washington SL, Herlemann A, Gomez SL, Carroll PR, Cooperberg MR (2021). Active surveillance in intermediate-risk prostate cancer with PSA 10–20 ng/mL: pathological outcome analysis of a population-level database. Prostate Cancer Prostatic Dis.

[13] Preisser F, Bandini M, Marchioni M, Nazzani S, Tian Z, Pompe RS, Fossati N, Briganti A, Saad F, Shariat SF, Heinzer H, Huland H, Graefen M, Tilki D, Karakiewicz PI (2018). Extent of lymph node dissection improves survival in prostate cancer patients treated with radical prostatectomy without lymph node invasion. Prostate.

[14] Al-Maghrabi JA, Bakshi NA, Farsi HM (2013). Gleason grading of prostate cancer in needle core biopsies: a comparison of general and urologic pathologists. Ann Saudi Med.

[15] Nagpal K, Foote D, Tan F, Liu Y, Chen PC, Steiner DF, Manoj N, Olson N, Smith JL, Mohtashamian A, Peterson B, Amin MB, Evans AJ, Sweet JW, Cheung C, van der Kwast T, Sangoi AR, Zhou M, Allan R, Humphrey PA, Hipp JD, Gadepalli K, Corrado GS, Peng LH, Stumpe MC, Mermel CH (2020). Development and validation of a deep learning algorithm for Gleason grading of prostate cancer from biopsy specimens. JAMA Oncol.

[16] Kvale R, Moller B, Wahlqvist R, Fossa SD, Berner A, Busch C, Kyrdalen AE, Svindland A, Viset T, Halvorsen OJ (2009). Concordance between Gleason scores of needle biopsies and radical prostatectomy specimens: a population-based study. BJU Int.

[17] Maruyama Y, Sadahira T, Araki M, Mitsui Y, Wada K, Rodrigo AGH, Munetomo K, Kobayashi Y, Watanabe M, Yanai H, Watanabe T, Nasu Y (2020). Factors predicting pathological upgrading after prostatectomy in patients with Gleason grade group 1 prostate cancer based on opinion-matched biopsy specimens. Mol Clin Oncol.

[18] van Leenders G, van der Kwast TH, Grignon DJ, Evans AJ, Kristiansen G, Kweldam CF, Litjens G, McKenney JK, Melamed J, Mottet N, Paner GP, Samaratunga H, Schoots IG, Simko JP, Tsuzuki T, Varma M, Warren AY, Wheeler TM, Williamson SR, Iczkowski KA, Members IGWP (2020) The 2019 International Society of Urological Pathology (ISUP) Consensus Conference on Grading of Prostatic Carcinoma. Am J Surg Pathol 44:e87-e99. 10.1097/PAS.0000000000001497 10.1097/PAS.0000000000001497 PMC7382533 32459716

[19] Varma M, Delahunt B, Egevad L, Samaratunga H, Kristiansen G (2019). Intraductal carcinoma of the prostate: a critical re-appraisal. Virchows Arch.

[20] (2016) WHO classification of tumours of urinary system & male genital organs. World Health Organization, Lyon, France

[21] Epstein JI, Egevad L, Amin MB, Delahunt B, Srigley JR, Humphrey PA, Grading C (2016) The 2014 International Society of Urological Pathology (ISUP) Consensus Conference on Gleason grading of prostatic carcinoma: definition of grading patterns and proposal for a New grading system. Am J Surg Pathol 40:244–252. 10.1097/PAS.0000000000000530 10.1097/PAS.0000000000000530 26492179

[22] Okubo Y, Sato S, Osaka K, Yamamoto Y, Suzuki T, Ida A, Yoshioka E, Suzuki M, Washimi K, Yokose T, Kishida T, Miyagi Y (2021). Clinicopathological analysis of the ISUP grade group and other parameters in prostate cancer: elucidation of mutual impact of the various parameters. Front Oncol.

[23] Hansel DE (2021). A 25 year perspective on advances in the pathologic assessment and diagnosis of urologic cancers. Urol Oncol.

[24] Paner GP, Stadler WM, Hansel DE, Montironi R, Lin DW, Amin MB (2018). Updates in the eighth edition of the tumor-node-metastasis staging classification for urologic cancers. Eur Urol.

[25] Dere Y, Celik OI, Celik SY, Ekmekci S, Evcim G, Pehlivan F, Agalar A, Deliktas H, Culhaci N (2020). A grading dilemma; Gleason scoring system: Are we sufficiently compatible? A multi center study. Indian J Pathol Microbiol.

[26] Allsbrook WC, Mangold KA, Johnson MH, Lane RB, Lane CG, Amin MB, Bostwick DG, Humphrey PA, Jones EC, Reuter VE, Sakr W, Sesterhenn IA, Troncoso P, Wheeler TM, Epstein JI (2001). Interobserver reproducibility of Gleason grading of prostatic carcinoma: urologic pathologists. Hum Pathol.

[27] Varma M (2021). Intraductal carcinoma of the prostate: a guide for the practicing pathologist. Adv Anat Pathol.

[28] Hoekstra RJ, Goossens WJH, Beulens A, van Herk H, Hoevenaars BM, de Baaij J, Somford DM, Sedelaar JPM, van Basten JA, Vrijhof H (2021). Reassessment of prostate biopsy specimens for patients referred for robot-assisted radical prostatectomy rarely influences surgical planning. Eur Urol Open Sci.

[29] Nakai Y, Tanaka N, Shimada K, Konishi N, Miyake M, Anai S, Fujimoto K (2015). Review by urological pathologists improves the accuracy of Gleason grading by general pathologists. BMC Urol.

[30] Ceylan C, Doluoglu OG, Aglamis E, Baytok O (2014). Comparison of 8, 10, 12, 16, 20 cores prostate biopsies in the determination of prostate cancer and the importance of prostate volume. Can Urol Assoc J.

